# Phycobilisome truncation causes widespread proteome changes in *Synechocystis* sp. PCC 6803

**DOI:** 10.1371/journal.pone.0173251

**Published:** 2017-03-02

**Authors:** Michelle Liberton, William B. Chrisler, Carrie D. Nicora, Ronald J. Moore, Richard D. Smith, David W. Koppenaal, Himadri B. Pakrasi, Jon M. Jacobs

**Affiliations:** 1 Department of Biology, Washington University, St. Louis, Missouri, United States of America; 2 Environmental Molecular Sciences Laboratory, Pacific Northwest National Laboratory, Richland, Washington, United States of America; 3 Biological Sciences Division, Pacific Northwest National Laboratory, Richland, Washington, United States of America; CEA-Saclay, FRANCE

## Abstract

In cyanobacteria such as *Synechocystis* sp. PCC 6803, large antenna complexes called phycobilisomes (PBS) harvest light and transfer the energy to the photosynthetic reaction centers. Modification of the light harvesting machinery in cyanobacteria has widespread consequences, causing changes in cell morphology and physiology. In the current study, we investigated the effects of PBS truncation on the proteomes of three *Synechocystis* 6803 PBS antenna mutants. These range from the progressive truncation of phycocyanin rods in the CB and CK strains, to full removal of PBS in the PAL mutant. Comparative quantitative protein results revealed surprising changes in protein abundances in the mutant strains. Our results showed that PBS truncation in *Synechocystis* 6803 broadly impacted core cellular mechanisms beyond light harvesting and photosynthesis. Specifically, we observed dramatic alterations in membrane transport mechanisms, where the most severe PBS truncation in the PAL strain appeared to suppress the cellular utilization and regulation of bicarbonate and iron. These changes point to the role of PBS as a component critical to cell function, and demonstrate the continuing need to assess systems-wide protein based abundances to understand potential indirect phenotypic effects.

## Introduction

Photosynthetic organisms use antenna systems to capture light energy as the first steps in converting sunlight and CO_2_ into bioproducts. In cyanobacteria such as *Synechocysti*s sp. PCC 6803 (*Synechocystis* 6803), the membrane protein complexes of the photosynthetic electron transfer chain reside in the specialized thylakoid membranes. To increase the amount of light energy transferred to reaction centers, cyanobacteria use large (3–7 MDa) phycobilisome (PBS) complexes that associate with the cytoplasmic surface of the thylakoid membrane and are composed of different pigment-proteins [1].

In *Synechocystis* 6803, PBS contain the biliproteins allophycocyanin (APC) and phycocyanin (PC), with a tri-cylindrical core composed of APC from which radiates an array of six PC rods. The PC rods are composed of three stacked PC hexamers attached by non-pigmented linker proteins [1]. In the current work, we used a series of three antenna mutants in which PBS are increasingly truncated. Specifically, the CB mutant lacks rod linker proteins and hence contains only a single PC hexamer per rod, for a total of six hexamers per phycobilisome [2]. The CB mutant was generated by disruption of the *cpcC1* and *cpcC2* genes [2]. The CK mutant was generated by disruption of the *cpcA* and *cpcB* genes (which encode the phycocyanin α and β subunits), as well as *cpcC1* and *cpcC2*. The CK mutant thus lacks all PC rod components and only contains the APC core [3]. The PAL mutant was generated by deletion of *apcE* (encoding the core-membrane linker) and *apcAB* (which encodes the allophycocyanin α and β subunits) in the PMB11 mutant background (which lacks phycocyanin). As a result, PAL lacks phycobilisomes entirely [4]. Studies using single particle electron microscopy have probed the structure of PBS from the CB and CK mutants at ~13 Å resolution, allowing the direct visualization of these truncated PBS [5]. Biochemical analyses have also examined the components of isolated complexes from PBS truncation mutants [2, 3, 6].

Besides genetic manipulation to modify PBS, cyanobacterial cells can modify the characteristics of PBS in response to changes in environmental conditions [7]. In the case of nitrogen or sulfur starvation, PBS degradation provides the opportunity for cellular recycling of nutrients contained in PBS, which due to their massive size, can constitute up to 50% of protein in the cell [8]. Recent studies have investigated the details of PBS degradation and the nature of the proteolytic process [9–11].

A number of studies have probed the effects of PBS modifications in cyanobacterial cells. Cell morphology is altered in PBS mutants, with thylakoid spacing [12, 13] and membrane curvature [13] decreasing as PBS size decreases. PBS truncation results in changes in photosystem I and II (PSI and PSII) stoichiometry, with an increase in PSII relative to PSI [4, 12–14]. PBS mutants have been studied by small-angle neutron scattering, revealing unique reversible light-dark subcellular structural changes in the repeating distances in the thylakoid membrane system [15, 16]. PBS truncation mutants have also been probed under a variety of conditions to determine whether antenna truncation provides a productivity advantage [6, 17, 18].

Given the wide-ranging effects of PBS modification in cyanobacterial cells, it is important to understand the underlying changes to the expressed proteins in the PBS truncation mutants. Furthermore, understanding in detail the cellular effects of phycobilisome modification is necessary if such modifications are to be aimed at tuning light harvesting and increasing productivity. Global proteomics studies can reveal the overall consequences of PBS modification on cell physiology. Previous proteomics experiments examining membrane and soluble fractions isolated from the Olive (lacking PC) and PAL mutant strains identified 397 and 218 proteins, respectively, with significant changes relative to wild type (WT) [18]. We have dramatically expanded this characterized to the CB, CK, and PAL phycobilisome mutants using a systems-wide, isobaric labeling, in-depth quantitative proteomics analysis, in order to more fully elucidate the related protein complexes and pathways differentially regulated due to phycobilisome modification. Using this approach, we identified 841 proteins with dramatic changes in differential abundances among the mutant strains. We see that even minor changes in light harvesting capability have a cascade effect on specific cellular processes, escalating to the most severe PAL mutant, where the total loss of the phycobilisome structure imposes large alterations and modifications to cellular functions. These mutants are a unique resource for further investigation, as they provide the ability to link phycobilisome alterations in energy capture with subsequent direct and indirect downstream consequences in the cell.

## Materials and methods

### Culture conditions

All chemicals used for media and other experiments are from Sigma (St. Louis, MO) unless otherwise noted. *Synechocystis* 6803 cultures of WT, CB, CK, and PAL were initially inoculated into 20 ml of liquid TES-buffered BG11 medium [19] and grown for 7 days in a Sanyo Versatile Environmental Test Chamber at 30°C under 20 μmol photons m^-2^ s^-1^ white fluorescent light with constant shaking at 125 rpm. Due to the different growth rates among the strains, cultures were sub-cultured based on cell concentration. The concentration of *Synechocystis* 6803 cultures were determined by flow cytometry using a BD Influx Fluorescence Activated Cell Sorter (FACS, BD Biosciences, San Jose, CA). Upon harvesting, 100 ml of each sample was analyzed using the 488-nm laser excitation from a Sapphire LP laser (Coherent Inc., Santa Clara, CA) at 100 mW. Forward and side scatter were used to gate out cellular debris and values are recorded. Optimization and calibration of the FACS was performed before each analysis using 3 μm Ultra Rainbow Fluorescent Particles (Spherotech, Lake Forest, IL). Cultures were then grown for 5 days, and harvested by centrifugation for proteomics analysis. Growth medium was supplemented with antibiotics as follows: CB and CK, 10 μg/ml kanamycin; PAL, 10 μg/ml chloramphenicol and spectinomycin [20].

### Sample preparation

0.5 mL of 0.5M TEAB was added to cell pellets with bead beating in a Bullet Blender (Next Advance, Averill Park, NY) at speed 8 for 3 minutes, at 4°C, then the lysate was spun into a falcon tube at 2000 x g for 10 minutes at 4°C. The sample was transferred into a fresh, labeled 5ml Eppendorf tube and ice cold (-20°C) chloroform:methanol mix (prepared 2:1 (v/v)) was added to the sample in a 5:1 ratio over sample volume and vigorously vortexed. The sample was then placed on ice for 5 minutes and vortexed for 10 seconds followed by centrifugation at 10,000 x g for 10 minutes at 4°C. The upper, water soluble phase was removed and 1 ml of ice cold methanol was added to precipitate the protein. The sample was again centrifuged at 10,000 x g for 10 minutes to pellet the protein and the methanol decanted off. The remaining protein pellet was placed in a fume hood to dry. Protein pellets were resuspended in 30 μl Universal Protein Extraction (UPX) buffer (Expedeon, San Diego, CA), and were sonicated, vortexed and incubated at 95°C for 5 minutes to reduce and denature the protein. The samples were allowed to cool at 4°C followed by centrifugation at 10,000 x g for 10 minutes. Amicon Ultra-0.5 ml 30k MWCO centrifuge devices (Millipore, Billerica, MA) were used for Filter Aided Sample Preparation (FASP)[21]. The sample was added to the filter unit with urea (8 M, pH 8.5 made with 0.5 M triethylammonium bicarbonate (TEAB)) at 15% of the total volume. The unit was centrifuged at 14,000 x g for 30 minutes. Alkylation was performed by adding 100 μl of 40 mM iodoacetamide in 0.5M TEAB and incubating at room temperature in the dark for 15 minutes. The sample was then spun at 14,000 x g for 15 minutes. Another 400 μl of urea was added and spun at 14,000 x g for 20 minutes and this was repeated 2 more times followed by 2 rinses with 0.5 M TEAB.

The spin filters were then transferred into fresh tubes and the sample was tryptically digested with Sequencing Grade Modified Trypsin (Promega) at 1:50 enzyme-to-substrate ratio and allowed to incubate overnight at 37°C. The following day peptide sample was centrifuged 14,000 x g for 15 minutes and the peptides were collected in the flow through. SPE C-18 columns (SUPELCO Discovery) were used for clean-up of the resultant peptide mixture, samples were concentrated down in SpeedVac SC250 Express (ThermoSavant) followed by a bicinchoninic acid assay (BCA assay) (Thermo Scientific, Rockford, IL) to determine final peptide concentration.

### iTRAQ labeling and HPLC fractionation

Isobaric labeling of captured peptides to achieve relative quantification using four-plex iTRAQ™ reagents was performed according to the manufacturer’s instructions (AB Sciex, Foster City, CA). For iTRAQ™ labeling, 30 μg of peptide for each time point was resuspended in 10.0 μl of dissolution Buffer (500 mM TEAB) mixed with the individual iTRAQ™ reagent and incubated at RT for 1 h. The reaction was stopped, and unreacted iTRAQ reagents were hydrolyzed by adding 150 μl of H_2_O and incubated at RT for 30 min. Contents of each iTRAQ reagent-labeled sample were then pooled, followed by concentration to 100 μl prior to HPLC fractionation.

Labeled peptide samples were separated at flow rate at 0.5 ml/min on a reverse phase Waters XBridge C18 column (250 mm × 4.6 mm column containing 5-μm particles, and a 4.6 mm × 20 mm guard column) using an Agilent 1200 HPLC System equipped with a quaternary pump, degasser, diode array detector, Peltier-cooled auto-sampler and fraction collector (both set at 4°C). 120 μg of labeled tryptic peptides was suspended in buffer A (10 mM TEAB, pH 7.5) and loaded onto the column. After the sample loading, the C18 column was washed for 35 min with solvent A, before applying the LC gradient. The LC gradient started with a linear increase of solvent A to 10% B (10 mM TEAB, pH 7.5, 90% acetonitrile) for 10 min, then linearly increased to 20% B at 15 min, 30 min to 30% B, 15 min to 35% B, 10 min to 45% B and another 10 min to 100% solvent B. Using an automated fraction collector, 96 fractions were collected for each sample, lyophilized and reconstituted into 12 fractions prior to LC-MS/MS analysis. These 12 final fractions were the result of concatenation through combining 8 fractions each, separated by 12 fractions apart.

### LC-MS/MS analysis

All iTRAQ™-labeled fractions were analyzed by LC–MS/MS. Each sample was loaded onto a Waters nano-Acquity dual pumping UPLC system (Milford, MA) custom configured for on-line trapping of 5 μl injection at 3 μl/min with reverse direction elution onto the analytical column at 300 nl/min. Columns were packed in-house using 360 μm o.d. fused silica (Polymicro Technologies Inc., Phoenix, AZ) with 1-cm sol-gel frits for media retention [22] and contained Jupiter C18 media (Phenomenex, Torrence, CA) in 5 μm particle size for the trapping column (150 μm i.d. x 4 cm long) and 3μm particle size for the analytical column (75 μm i.d. x 70 cm long). Mobile phases consisted of (A) 0.1% formic acid in water and (B) 0.1% formic acid in acetonitrile with the following gradient profile (min, %B): 0, 1; 2, 8; 20, 12; 75, 30; 97, 45; 100, 95; 110, 95; 115, 1; 150, 1.

MS analysis was performed using a LTQ Orbitrap Velos mass spectrometer (Thermo Scientific, San Jose, CA) outfitted with a home-made nano-electrospray ionization interface using 150 um o.d. x 20 μm i.d. chemically etched fused silica [23]. The heated capillary temperature and spray voltage were 350°C and 2.2 kV, respectively. Data were collected for 100 min following a 15 min delay from sample injection. FT-MS spectra were acquired from 300–1800 m/z at a resolution of 30k and while the top 10 FT-HCD-MS/MS spectra were acquired in data dependent mode at a resolution of 7.5k using a normalize collision energy of 32.

### Data analysis

LC–MS/MS raw data were converted into dta files using Bioworks Cluster 3.2 (Thermo Fisher Scientific, Cambridge, MA, USA). The MSGF+ algorithm [24] was used to search MS/MS spectra against the *Synechocystis* 6803 database (NCBI 2011-02-28, 3672 entries). The key search parameters used were 20 ppm tolerance for precursor ion masses, +2.5 Da and -1.5 Da window on fragment ion mass tolerances, no limit on missed cleavages, partial tryptic search, no exclusion of contaminants, dynamic oxidation of methionine (15.9949 Da), static IAA alkylation on cysteine (57.0215 Da), and static iTRAQ modification of lysine and N-termini (+144.1021 Da). The decoy database searching methodology [25, 26] was used to control the false discovery rate at the unique peptide level to <0.01% and subsequent protein level to <0.1% [24]. Quantification was based upon specific peptide reporter ion intensities captured across all channels and compared by calculating the summed peptide intensity values across fractions for each respective sample type (WT, CB, CK, and PAL). Only proteins containing multiple peptide identifications with reporter ion intensities were quantified. The mass spectrometry proteomics data have been deposited to the ProteomeXchange Consortium [27] via the PRIDE partner repository with the dataset identifier PXD003023 and 10.6019/PXD003023.

### Experimental design and statistical rationale

**Fig 1:**
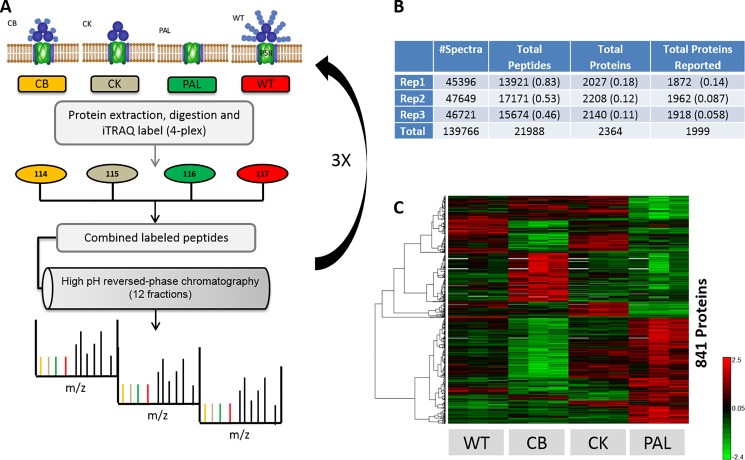
Overview of proteomic analysis and initial results. A) Schematic design of iTRAQ based quantitative LC-MS/MS experiment for comparison of each PBS mutant against WT. Triplicate biological replicates, iTRAQ experiments, were performed based upon off-line high pH reversed phase peptide level separations prior to LC-MS/MS analysis of each fraction. B) Table providing total peptide and protein level identification after data processing using MSGF+. Numbers in parenthesis represents % standard deviation of results across channels per biological replicate. C) Heat map of proteins after ANOVA statistical comparison (pval <0.01) for all conditions, demonstrating biological replicate consistency and extensive differential protein abundances across PB mutants.

Fig 1 outlines the experimental design with triplicate biological replicates (iTRAQ 4) of the three *Synechocystis* 6803 mutants and WT controls (total of 12 samples). Peptide reporter ion intensities were summed within protein/experiment, median central tendency normalized, and then average reference scaled within iTRAQ experiment. Statistical comparison was based upon a simple one-way ANOVA with mutant sample type as a fixed effect using the program DAnTE [28]. Confidence criteria used are as specified in the results.

## Results

### Protein identification in WT and mutant strains

The goal of this study was to provide a robust quantitative assessment of the cellular effects of PBS truncation in the CB, CK, and PAL mutant strains. Identification and quantification of the protein complement of *Synechocystis* 6803 required a large scale, whole proteome LC-MS/MS approach. The approach entailed a 4-plex iTRAQ labeling strategy across all mutant strains for comparison against WT, repeated for three biological replicates (Fig 1A and S1 Table). Post-labeling high pH fractionation coupled with LC-MS/MS analyses resulted in a comprehensive capture of >1900 proteins (summarized in Fig 1B) that provides the necessary scope for differential protein pathway/complex interpretation. An initial statistical comparison across all mutant conditions versus WT revealed that a large percentage of quantified proteins were deemed significant (pval <0.01) within some comparison (see Fig 1C). Using this analysis, we identified 841 proteins for which dramatic changes in differential abundances could be seen among the strains (Fig 1C).

### Phycobilisome protein characterization across mutants

**Fig 2:**
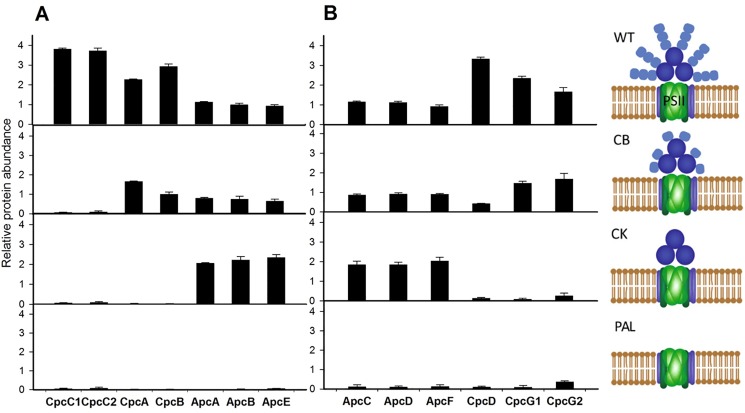
Comparison of phycobilisome structural protein abundances. A) Relative protein quantification of phycobilisome proteins directly knocked out in PBS mutant strains CB, CK, and PAL. B) Quantification of remaining phycobilisome structural proteins detected and measured. Error bars represent the standard deviation across biological triplicate analysis.

An initial quantitative assessment of phycobilisome proteins showed that measured protein abundances correlated well with the deleted genes in the mutant strains. Fig 2A shows the detected protein abundances of all deleted or attenuated phycobilisome complex proteins in the mutants compared to WT. The CpcC1 and CpcC2 PBS rod linker proteins are deleted in the CB mutant, resulting in PC rods with only one hexamer [2]. In the CK mutant, the entire *cpc* operon is deleted, eliminating all PC hexamers and leaving only the core APC components [3], and in the PAL mutant, the loss of PC, the ApcAB allophycocyanin chains, and ApcE core membrane linker essentially removes all functional phycobiliproteins [4]. In total, all explicitly deleted PBS proteins were observed with proteomic abundances at near zero or noise thresholds, as expected. Additional observations, however, include a marked increase in the remaining APC core proteins in CK compared to WT and CB mutants (Fig 2A), implying an increase in expression of APC in the CK mutant.

Fig 2B shows the relative protein abundance of the remaining PBS proteins that are products of genes that were not specifically deleted in any of the mutants. Phycocyanin rod-linker protein CpcD is present but dramatically reduced in CB, consistent with earlier findings showing that CpcD was present in isolated phycobilisomes [2]. CpcG1 and CpcG2 rod-core linker proteins in CB are similar to WT, but are absent in CK. APC core proteins (ApcD, ApcF, ApcC) are also observed in higher abundance for CK versus CB and WT, similar to ApcA, ApcB, and ApcE in Fig 2A. The PAL mutant is once again void of any remaining PBS proteins, with a slight possible detection of remnant CpcG2; however, this may not be actual protein abundance but just slightly elevated noise detection. Additionally of interest, CpcE and CpcF, phycocyanobilin lyase subunits, though not structurally associated with PBS, were clearly abundant across WT and all mutants, with the highest abundance observed in the PAL mutant (S1 Fig).

### Systems-wide mapping of altered proteins linked to phycobilisome truncation

**Fig 3:**
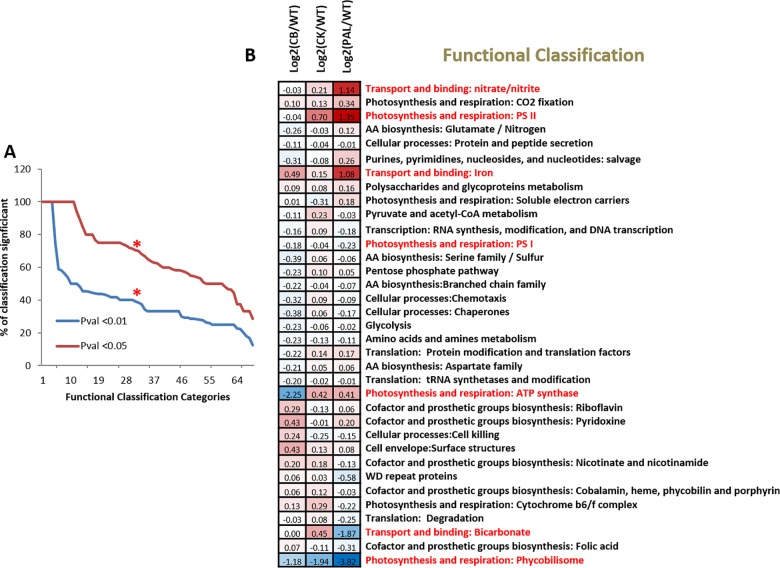
Functional classification comparisons of protein identifications. Mapping to Cyanobase gene function classification assigned functional categorizations to identified proteins. Relative protein abundances were then averaged within each classification and statistically compared to WT. A) Rank order chart of 69 functional classifications with significant protein abundance shifts showing % protein significance of each classification compared to WT. The red asterisk represents a cut-off of the 35 functional classifications that had at least 40% (pval <0.01) or 75% (pval <0.05) of the detected proteins as significantly altered due to PBS truncation. B) Table of the 35 classifications representing highly altered cellular functions, with mean protein abundance shift for each classification per PBS truncation relative to WT. Color coding shows red as increased abundance compared to WT and blue as decreased abundance compared to WT.

To understand the systemic effects that PBS truncation placed upon *Synechocystis* 6803 cellular functions, all quantitatively detected proteins were mapped for functional annotation using Cyanobase gene function categorization (see S2 Table). Out of 79 total functional categories containing multiple detected proteins, 69 categories had at least one protein with significant abundance shifts (pval <0.01) for any mutant. Fig 3A shows the distribution of all 68 functional categories based upon % of significant proteins. 35 functional classifications had at least 40% (at pval <0.01) or 75% (at pval <0.05) of the detected proteins as significantly altered due to PBS truncation. Fig 3B plots these 35 highly altered cellular functions and shows the mean protein abundance shift for each classification in each mutant relative to WT. Several cellular functions have large shifts in overall protein abundance as *Synechocystis* 6803 cells attempt to adjust cellular mechanisms in response to decreased light harvesting ability. These functional classifications include photosynthesis and respiration, as well as other less predictable categories such as transport and binding for iron, nitrate, and bicarbonate. Functional groups highlighted in red in Fig 3B are specifically discussed below.

### PSI/PSII protein ratio shifts in response to PBS truncation

**Fig 4:**
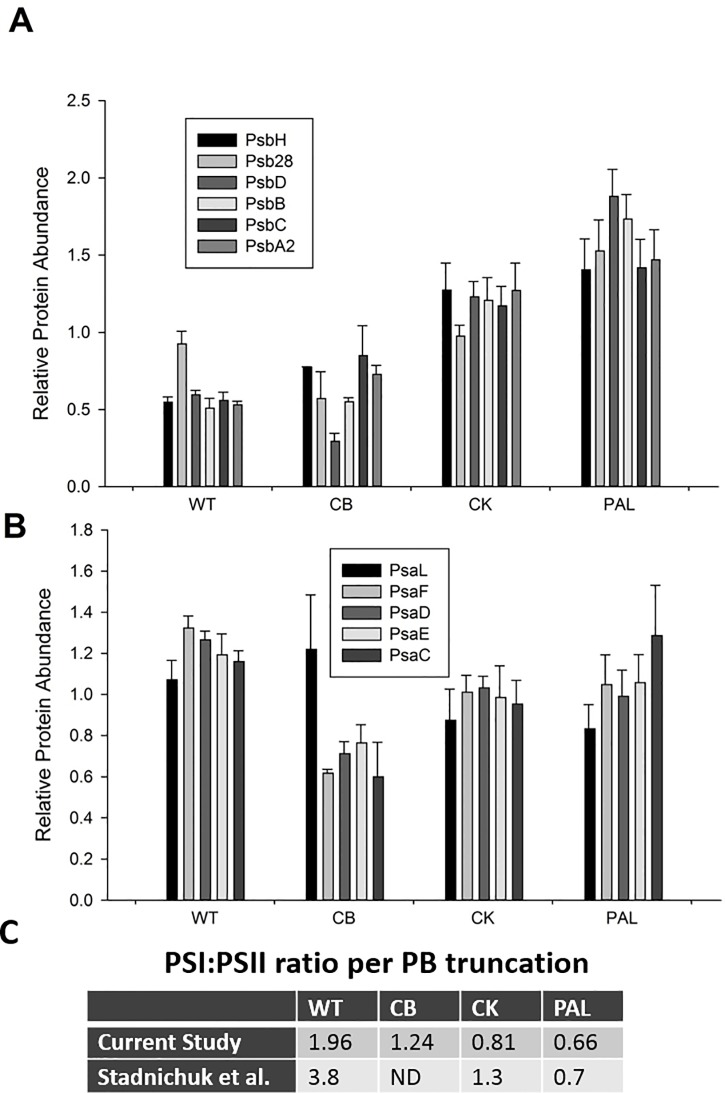
PSII and PSI subunit protein levels. A) Quantitative protein levels of PSII subunits showing increases in abundance with increasing truncation of phycobilisome structure. B) Quantitative protein levels of PSI subunits showing detectable but minimal reduced abundances due to PB truncation. Error bars represent standard deviation of biological triplicate measurements. C) Table summary of calculated PSI/PSII ratios of current study compared to previous observations.

As seen in Fig 4, both PSI and PSII protein levels are modified due to progressive PBS truncation. Fig 4A and 4B shows the distribution of the individual PSI and PSII protein abundances, where increasing truncation tracks with an increase in PSII levels (Fig 4A), coupled with a milder but consistent decrease in PSI protein abundances in the PBS mutant strains (Fig 4B). Interestingly, PsbD (the D2 reaction center protein) is in lower abundance in CB compared to WT, a result not seen in the CK or PAL strains, where PsbD is increased along with other PSII proteins (Fig 4A). PsaL, the PSI protein required for trimer formation [29], is found in higher abundance in CB compared to other PSI proteins (Fig 4B).

The respective PSI/II ratios are shown in Fig 4C, calculated based on the total detected intensity summed across all peptides for each respective protein, averaged across either PSI or PSII. Results are consistent with previous observations [4, 12, 14] and verify that increases in fluorescence measurements assigned to PSII are a direct effect of increasing PSII protein abundance in the mutant strains [30, 31]. We also concur [13] that the PS I/II ratio shift appears to be driven more by an increase in PSII compared to the more subtle decrease in PSI from PBS truncation (Fig 4B).

### Alterations in iron transport and regulation due to PBS truncation

**Fig 5:**
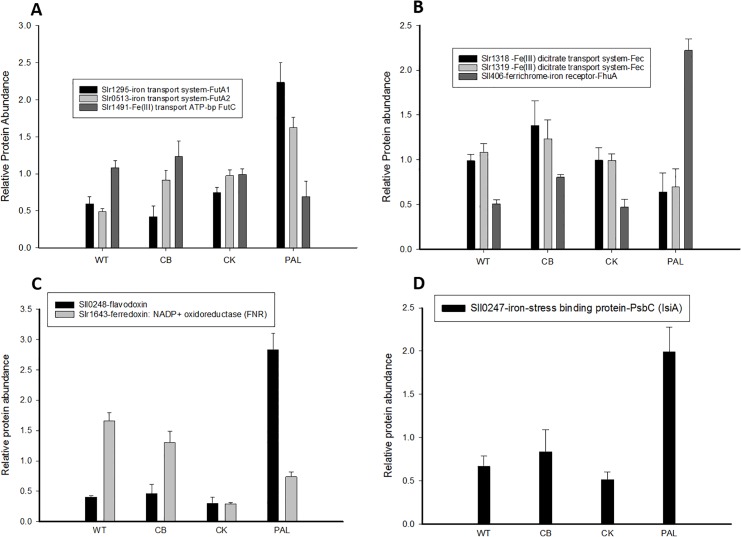
Changes in abundance of iron transport and response proteins. A) Protein abundances for the FutA1, FutA2, and FutC components of the (ABC)-type ferric iron transporter. B) Protein abundances of Fe(III) dicitrate transport system proteins showing a decrease in abundance primarily for PAL, compared to an increased abundance of FhuA in PAL. C) Change in the abundance of electron acceptors for photosynthesis, from the iron containing ferredoxin: NADP^+^ oxidoreductase (FNR) in WT to the iron lacking flavodoxin in PAL. D) Protein abundances for the iron stress-induced IsiA protein. Error bars represent standard deviation of biological triplicate measurements.

In cyanobacteria, iron is a limiting cofactor for growth and is required to maintain functional PSI complexes [32]. In aquatic environments, bioavailability of iron is typically low, in contrast to the high concentrations (~30 μM) in standard BG11 growth media [19]. Perturbations in abundance were observed for proteins involved in molecular transport and binding associated with iron (Fig 5). Specifically, iron binding/transport system proteins FutA1-A2, components of the FutABC transporter for inorganic Fe(III) [33], were higher in protein abundance upon complete PBS truncation in the PAL strain compared to WT, CB, and CK (Fig 5A). FutA2 was increased in both CB and CK, while FutA1 decreased slightly in CB compared to WT. This is in contrast to the FecB and FecE components of the iron (III) dicitrate transport system (Fig 5B), and FutC (Fig 5A), all of which showed a drop in protein abundance in PAL compared to the other strains (Fig 5B). Also found in high abundance in the PAL strain was FhuA, the receptor component involved in the transport of ferric siderophones [33] (Fig 5B).

The IsiA protein is expressed under iron starvation and other stress conditions in cyanobacterial cells [34] [35, 36]. IsiA was increased in the PAL mutant (Fig 5D), an unexpected result given that cells were grown in iron replete conditions. Additionally, we observed in CK and PAL a decrease in abundance of ferredoxin:NADP^+^ oxidoreductase (FNR), the enzyme that catalyzes the transfer of electrons between ferredoxin and NADP [3] (Fig 5C). This coincides with an increase in the abundance of flavodoxin (which can replace ferredoxin as a soluble electron carrier under iron deficient conditions) in PAL, although not in CK (Fig 5C). In comparison to previous studies, Kwon, et al., lacked sufficient quantitative data for IsiA and FNR comparisons, but similar results were noted in the increase in FutA1, FutA2, and flavodoxin in PAL [18].

### Changes in proteins involved in nitrogen assimilation

**Fig 6:**
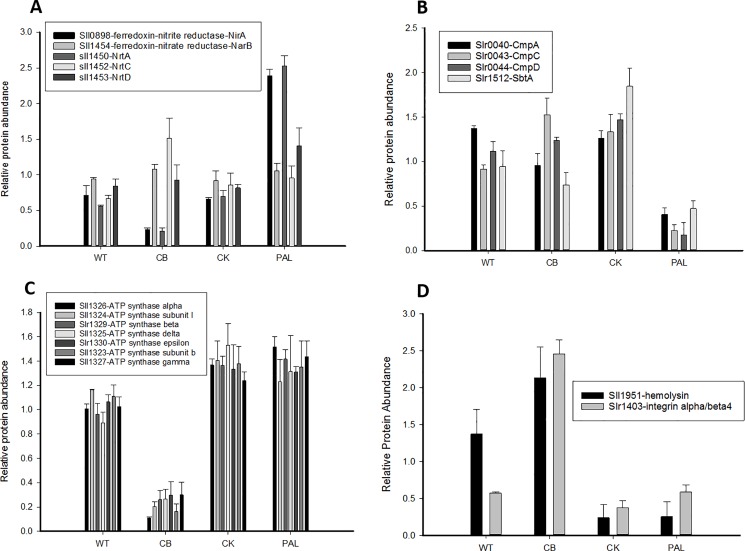
Additional significantly altered protein groups. A) Abundance of proteins involved in nitrogen assimilation. B) Protein abundances for bicarbonate transport mechanisms (CmpACD and SbtA). Extensive reduction in abundance is observed for the PAL mutant only. Protein Slr0041 (CmpB) showed a similar distribution pattern but was not included as it was only represented by 1 peptide for abundance calculations. C) Graph of protein abundance distribution comparing ATP synthase complex subunits. A large abundance decrease in the CB mutant is observed. D) Graph of the unique protein distribution of S-layer proteins hemolysin and integrin alpha/beta4, with a dramatic increase for the CB mutant. Error bars represent standard deviation of biological triplicate measurements.

We examined the abundances of proteins involved in nitrogen assimilation, in particular the components of the ABC-type transporter for nitrate uptake, NrtABCD, the ferredoxin-nitrate reductase NarB and the ferredoxin-nitrite reductase NirA (Fig 6A). NarB and NirA perform the two-step process of the reduction of nitrate to ammonium [37]. Interestingly, of these, NirA was much reduced in CB and dramatically increased in PAL (Fig 6A). Of the transporter components, NrtA abundances were similar to NirA, decreased in CB and increased in PAL (Fig 6A).

### Reduction of bicarbonate transport proteins in PAL clarifies sensitivity to pH

Previous physiological characterization of PBS truncation mutants demonstrated that the PAL strain could not survive at increasingly higher pH conditions (beginning at pH 10.3), unlike WT, CB, and CK cultures [17]. In the current analysis, bicarbonate transport proteins were observed as one of the most highly reduced categories in PAL (see Fig 3B, at -1.95). Fig 6A shows the detected individual components of the bicarbonate transport system (CmpABCD), as well as SbtA. We see extensive reduction in all these bicarbonate transporters in the PAL mutant, providing a likely mechanism for PAL high pH sensitivity. The additional bicarbonate uptake protein BicA (sll0834) was not detected quantitatively in this study, nor was any member of the CO_2_ uptake system NDH-I_3_, so it is unclear what contributions these systems could be making due to their limited abundance. We did, however, quantitatively identify three out of the four members of the CO_2_ uptake system NDH-I_4_ (sll1733-1735) (S2 Fig), though there appeared to be minimal change or too much variability between replicates to draw any conclusions as to the overall differential abundance with this system. Verification of these results in Kwon et al, was difficult as all *cmp* components and SbtA were either not detected or minimally identified [18].

### Unique ATP synthase alterations in CB

One of the more striking observations involved the ATP synthase complex, which was observed as significantly down regulated in only CB (Fig 6B). Considering that each subunit was identified by between 13–50 individual peptides, we are confident in the overall identification and quantitation detected. Interestingly, it is well recognized that the growth and physiological characteristics of CB are the most similar to WT among the three PBS truncation mutants; hence it appears that CB is able to overcome any deficiency in ATP synthase. Such a finding implies a possible connection between the specific CB PBS truncation and ATP synthase, with some unique aspect of the minimal PB truncation helping drive a decrease in ATP synthase structure abundance.

### Alterations in cell surface proteins

In our analysis, we identified two differentially abundant cell surface proteins, Sll1951-hemolysin [38] and Slr1403-integrin alpha/beta4 [39] (Fig 6D). Sll1951-hemolysin appears increased in CB only, with dramatic reductions in protein levels for CK and PAL. A recent study showed that the deletion of *sll1951* resulted in cells lacking the characteristic surface layer (S-layer) pattern as the outermost cell surface, thus identifying Sll1951 as the S-layer protein [38]. However, previous morphological studies of PBS mutants using TEM did not detect any gross alterations in the cell envelope [15]. Additionally, integrin alpha/beta4 abundance appears to mirror hemolysin except for the baseline reduction in WT values. Neither hemolysin or integrin alpha/beta4 was identified in Kwon et al. with enough robustness to compare quantitatively [18].

## Discussion

While initially investigated as an approach to understanding phycobilisome structure, with implications towards designing more efficient antenna, *Synechocystis* 6803 PBS truncation mutants [2, 4, 5] have now been subject to multiple characterizations revealing insights into membrane organization, morphology, physiology, and growth characteristics [13, 15, 17, 40, 41]. Now, after extensive proteomic quantitative characterization, we have greater insight into specific previously observed physiological behaviors and a more thorough understanding of the systemic impact of these truncations on cellular mechanisms, many reaching well beyond the direct associations with PSI/PSII energy capture and conversion to cellular fuel. Previous proteomic studies by Kwon et al. using mutant strains PAL and CK help support the results for those proteins that were previously quantitatively identified; however, the additional coverage and quantitative information provided in the current study allowed for a more comprehensive view of the proteome.

Investigating the proteome of the phycobilisome structure itself, observations were largely as expected, with clear quantitative values corresponding to the deletion of all relevant PBS truncation proteins (Fig 2). Additionally, phycocyanin proteins in CK and all phycobilisome proteins in PAL expressed from genes that were not explicitly deleted were also absent in these mutants. The only exception to this observation was CpcD in CB, which was attenuated in protein abundance.

The diverse and widespread functional alterations observed in Fig 3 confirm that systematic perturbations to cellular pathways and functions extend well beyond a direct influence on phycobilisome light capture. Based upon past studies [13, 30], we looked specifically at possible alterations to PSII and PSI levels. The measured changes in PSI/II protein levels were similar to calculations based on 2D images and fluorescent measurements [13], and our data confirm that this is driven primarily by increases in PSII levels that are proportional to the severity of the PBS truncation, with a concurrent but a minimal decrease in PSI. Although we recognize that protein quantification of specific PSII and PSI subunits does not necessarily represent active complexes, the general quantitative consistency of the subunits for each PBS truncation provides a strong rationale for interpreting these finding as representative of active protein components. Interestingly, we also see significant shifts in the abundance of the cytochrome *b*_*6*_*f* complex (see Fig 3) with general protein abundance increases in the CB and CK strains; however, PAL shows a decrease in *b*_*6*_*f* complex protein levels. Taken together, with our understanding of PSII and PSI spatial localization, altered morphology, and diverse alterations across PBS truncation variants, it is clear that each truncation has in many instances produced unique and often unpredictable perturbations. This is also apparent when looking at the abundance profile of another thylakoid membrane complex, ATP synthase (Fig 6C). The reduced protein signature of ATP synthase for CB remains puzzling. In cyanobacterial cells, the proton influx from the cytoplasm into the thylakoid lumen due to the activity of the photosynthetic electron transport chain and respiration must be balanced with proton efflux via ATP synthesis [42]. How this is accomplished in the CB mutant with lower levels of the ATP synthase protein components is unknown and surprising, particularly since the CB mutant is the most similar to WT in terms of growth characteristics and cell morphology. Nevertheless, it is not surprising that there are unforeseen alterations to integral thylakoid membrane components. It is unlikely that this observation is a quantitative artifact because comparisons were based upon total protein per sample, with only minor global abundance shifts observed (primarily for PAL) with subsequent minimal correction (see Materials and Methods). Additionally, we did not detect any major shifts for CB in other membrane protein components (PSI/PSII) or globally (see S3 Fig for summary comparison of all ribosomal 30S and 50S protein components).

There also were variations in proteins associated with transport and binding of small molecules/ions (Figs 5 and 6). We looked closely at iron and bicarbonate associated transport mechanisms, and found that perturbations caused by PBS truncation, particularly in PAL, altered the cellular state into a profile similar to iron depletion. The increased abundance of FhuA, FutA1 and FutA2 proteins showed similar abundance profiles with previous iron depletion/repletion studies [43]. Additionally, the IsiA protein [35, 36], a hallmark iron stress CP43 homolog, was also increased in PAL. IsiA, through its association with PSI, is proposed to increase the overall absorption cross-section and light harvesting capability of PSI [44]. This increased abundance of IsiA in the PAL strain may be a strategy for the cells to compensate for decreased light harvesting capability due to lack of PBS. Furthermore, we observed flavodoxin (IsiB) as also highly increased in abundance in PAL, marking the shift away from the iron-containing electron acceptor, ferredoxin (FNR), to the iron-less flavodoxin receptor (Fig 5C). Previous iron depletion/repletion studies showed complementary large increases in IsiB [43]. However, the presence of IsiA, and the corresponding blue shift in the room temperature chlorophyll *a* absorbance peak from 680 nm to 673 nm [45], has not previously been found in the PAL strain [4, 13]. We do note that even though IsiA does increase in relative abundance at least 2-fold over basal WT levels, IsiA is still in relative low abundance in the current dataset compared to the levels detected in previous iron depletion/repletion studies [43]. We have observed a small shift in the chlorophyll absorbance peak to 677 nm for PAL that might be due to the presence of IsiA (data not shown), but additional experiments will be necessary to confirm this finding. The question remains as to whether the lack of PBS intrinsically induces the iron stress response system, or if it could be driven by the increase in overall PSII complex abundance and lower levels of PSI, requiring higher light harvesting capability by PSI. Alternately, IsiA may be produced in PAL as a stress response to the cell-wide disequilibrium caused by lack of PBS. Further studies are needed to answer this question in its entirety.

Large changes were also noted in the PAL strain when components of the nitrogen assimilation system were examined (Fig 6A), with abundance increases found particularly in NirA and NrtA. Likewise, bicarbonate transport proteins showed a significant decrease in protein abundance exclusively in PAL, which we have functionally linked to this strain’s inability to manage bicarbonate levels at elevated pH. What then underlies the inability of PAL to increase functional bicarbonate transport remains an open question.

The concept of reducing the photosynthetic light harvesting antenna to prevent excess absorption of photons under bright light conditions has been proposed as a method of increasing productivity for biotechnological applications [41, 46–48]. Several studies have concluded that the PAL mutant, with complete PBS truncation, is not a good candidate for such applications due to its poor growth and lack of robustness [6, 17, 18]. Studies using, in particular, less severe PBS truncation mutants have shown an advantage to PBS truncation under optimized conditions [18, 41]. The results of this proteomics analysis show that PBS truncation has far-reaching and unpredictable consequences and therefore warrants particular care when used to evaluate criteria such as productivity advantages due to the metabolic tradeoffs that occur in these strains.

Overall, the message conveyed by protein quantitation supports the conclusion that PBS truncation in *Synechocystis* 6803 dramatically alters core cellular mechanisms beyond energy capture and electron transport, and as such, the constraints placed upon molecular processes dramatically alter protein abundances and hence the phenotype of the mutant strains. The most dramatic alterations appear localized within membrane associated functions and regulation of cellular resources (i.e., iron, nitrite/nitrate, bicarbonate), and interestingly are both directly and indirectly associated with the truncation. Finally, each PBS truncation, though progressive in nature, often exhibited a unique phenotypic compare to WT. We assert that in the current realm of extensive bioengineering and bio-design for more amenable energy producing organisms, there remains a continuing need to assess the systems-wide, protein based implications of even seemingly minor alterations to the system.

## Supporting information

S1 Fig Bar graph of non-structural phycobilisome associated protein abundances for CpcE and CpcF. Error bars represent standard deviation of triplicate biological replicates.
(JPG)

S2 Fig Bar graph of the detected and quantified components of the CO_2_ uptake complex NDH-I_4_. Error bars represent standard deviation of triplicate biological replicates.
(JPG)

S3 Fig Bar graph of the average protein abundance values of 30S and 50S ribosomal proteins. Error bars represent standard deviation across all 30S (21) and 50S (30) proteins.
(JPG)

S1 Table Peptide level identifications and quantification values based upon iTRAQ reporter ions. (XLSX)

S2 Table Protein level identifications and quantification values. (XLSX)
