# Supplementary figures and images for: Phycobilisome truncation causes widespread proteome changes in Synechocystis sp. PCC 6803
Source: PLoS One. 2017 Mar 2;12(3):e0173251. doi: 10.1371/journal.pone.0173251 (PMC5333879; doi:10.1371/journal.pone.0173251)

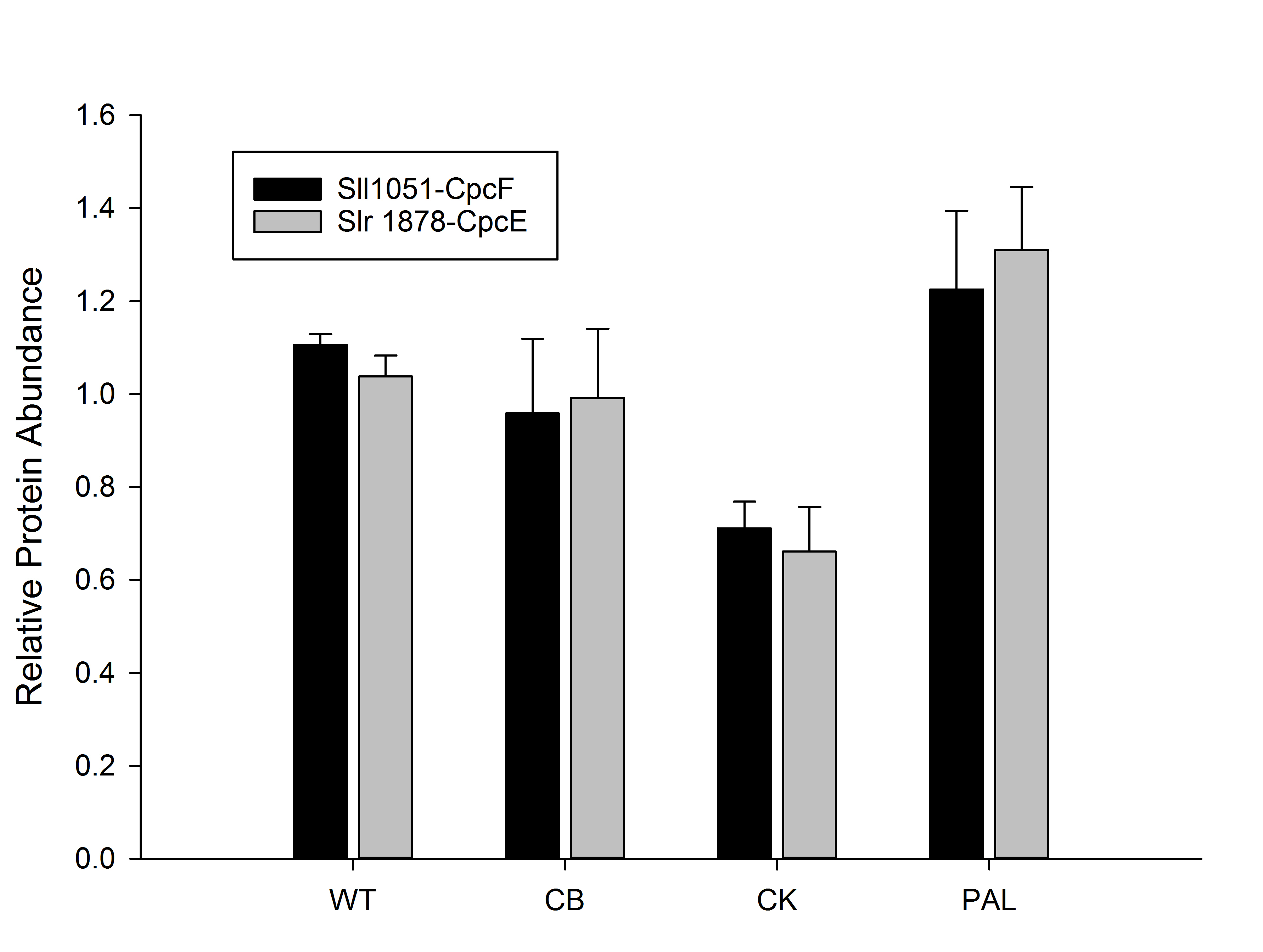

Supplement: S1 Fig — Error bars represent standard deviation of triplicate biological replicates. (JPG) [file pone.0173251.s001.JPG]

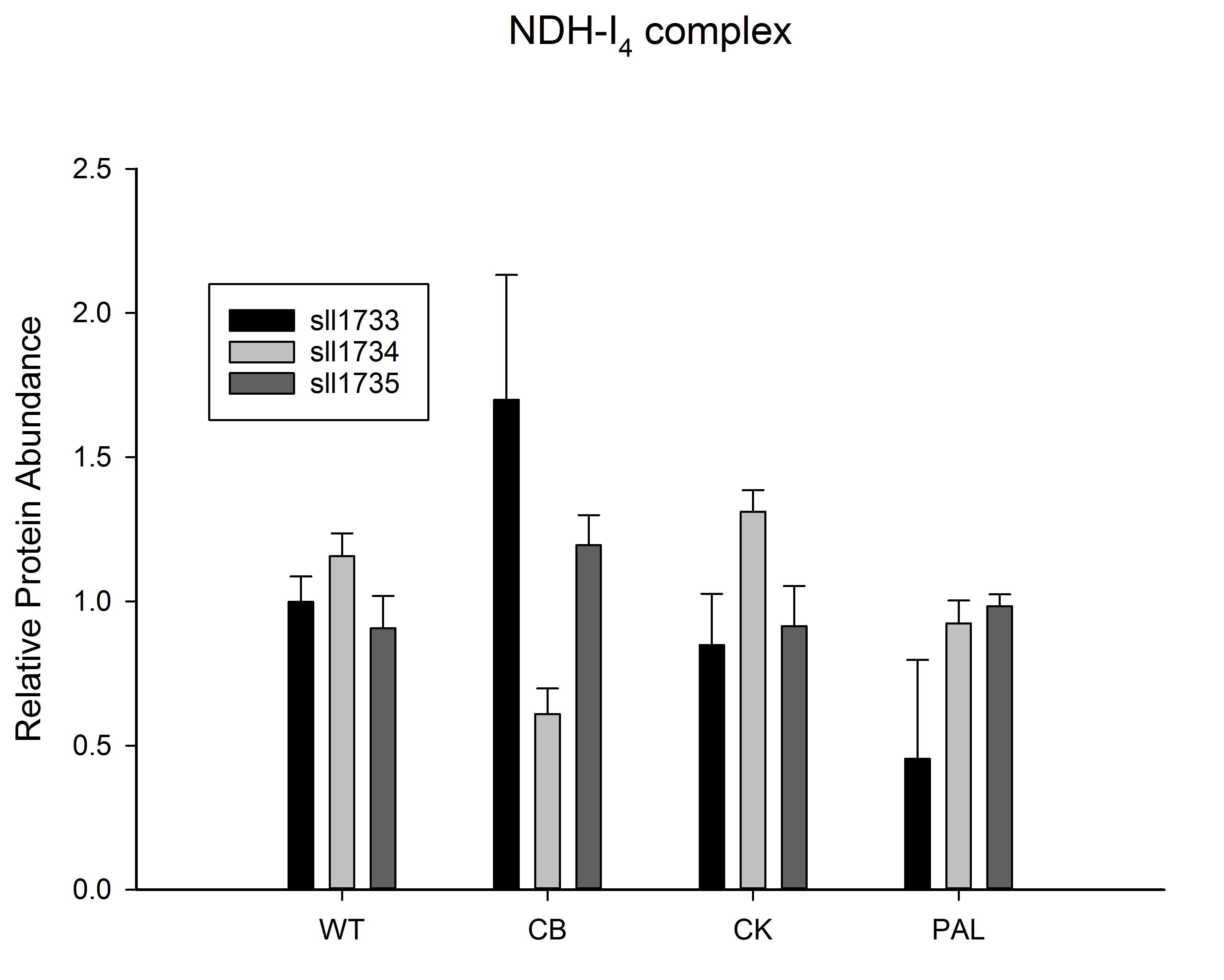

Supplement: S2 Fig — Error bars represent standard deviation of triplicate biological replicates. (JPG) [file pone.0173251.s002.JPG]

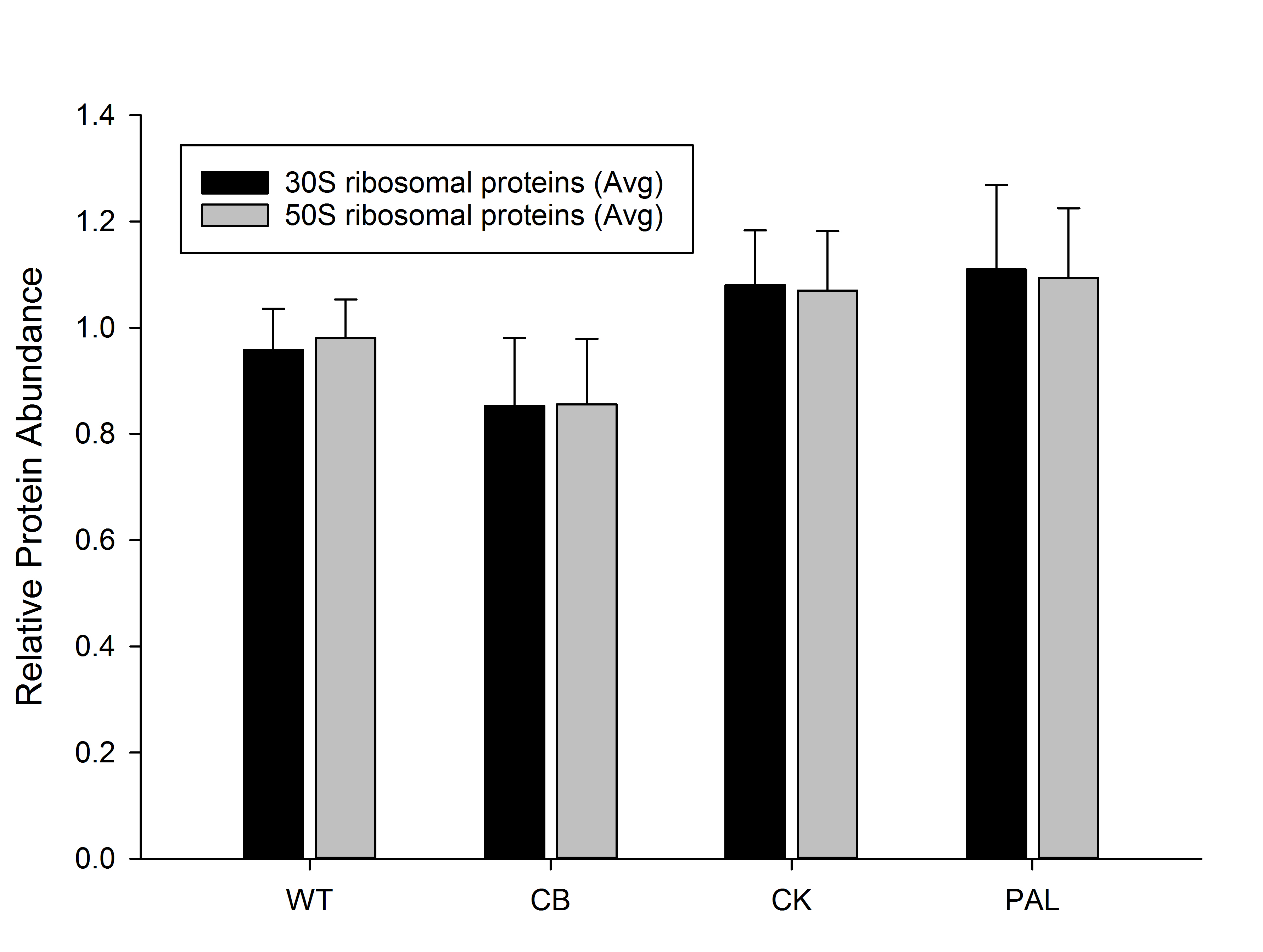

Supplement: S3 Fig — Error bars represent standard deviation across all 30S (21) and 50S (30) proteins. (JPG) [file pone.0173251.s003.JPG]
